# Use of hospital-based health care services among children aged 1 through 9 years who were born very preterm - a population-based study

**DOI:** 10.1186/s12913-017-2498-3

**Published:** 2017-08-17

**Authors:** Søren T. Klitkou, Tor Iversen, Hans J. Stensvold, Arild Rønnestad

**Affiliations:** 1 Department of Health Management and Health Economics, University of Oslo, P.b. 1089 Blindern, NO 0317 Oslo, Norway; 2 Clinic for Pediatric and Adolescent Medicine, Neonatal Intensive Care Unit, Oslo University Hospital, Rikshospitalet, Oslo, Norway; 3 Institute for Clinical Medicine, University of Oslo, Oslo, Norway

**Keywords:** Preterm, Morbidity, Long-term outcomes, Hospitalization, Gestational age

## Abstract

**Background:**

Very preterm (VPT) children, with a birth weight below 1500 g or delivered before 32 weeks of gestational age, are at increased risk of poorer long-term health outcomes and higher rates of hospitalization in childhood. However, considerable variation exists in the need for in-hospital care within this population. We assessed the utilization and distribution of hospital-based care from ages 1 through 9 years for a nationwide population.

**Methods:**

This was a population-based cohort of VPT children born in the period 2001–2009. We evaluated their utilization of hospital care in 2008–2010, when aged 1–9 years old. Outcomes were the incidence of hospital admissions and outpatient visits. We used Poisson regression models with multiple imputation of missing data.

**Results:**

Children born VPT had more hospital admissions compared with the general population of children aged 1–9 years. The rates of hospital admissions and outpatient visits were strongly related to clinical characteristics of the child at birth and age at admission/outpatient visit but to only a variable and minor degree to characteristics pertaining to maternal health, the sociodemographic factors, and geographical proximity to hospital services.

**Conclusions:**

Prior to this study, hospital utilization during the period 5–9 years old has been poorly documented. We found that excess utilization of hospital resources on average declines with increasing age. We also noted substantial differences in the use of hospital care across age groups and clinical factors for VPT children. The added information from the health status of mothers, social background, and geographic measures of access was limited.

**Electronic supplementary material:**

The online version of this article (doi:10.1186/s12913-017-2498-3) contains supplementary material, which is available to authorized users.

## Background

Children with a birth weight (BW) below 1500 g or delivered before 32 weeks of gestational age (GA) — henceforth referred to as very preterm (VPT) children — require considerable care in the neonatal period, and are at significant risk of long-term sequelae [1–4]. The initial frailty of these children leads to poorer long-term health and social outcomes compared with children delivered at term [5–8]. Long-term consequences have been investigated by Moster et al. [7], who described associations between levels of GA and a variety of medical and social outcomes later in childhood and young adulthood. Among these outcomes were the frequency of cerebral palsy, poor schooling outcomes, and retirement from working life due to disability.

As modern treatment increases survival at increasingly lower levels of GA, the surviving children may have higher levels of morbidity for which they require care later in life, thus studies with long-term follow-up of care provision are increasingly important to undertake [1, 9]. Even if the frequency of treatment within the tertiary care sector varied according to the characteristics of the health care system, prematurity has been shown to lead to excess hospitalizations at later stages in childhood, across a range of health care systems [10–20].

Generally, previous studies have emphasized how variations in the use of hospital services arise predominantly in relation to GA and clinical characteristics. However, geographic, sociodemographic, and socioeconomic characteristics are known to play a role in children’s health and their utilization of health care services [8, 21]. For decision makers concerned with the long-term health outcomes associated with prematurity, monitoring the variation in hospital use can provide an indication of the persistence of initial disadvantageous clinical characteristics. Furthermore, it can serve as an indication of whether differences exist due to characteristics extraneous to clinically evaluated need [22]. The decision maker may consider expectations of equal treatment for equal need across socioeconomic and geographic characteristics relevant when considering the underlying need for care of this population.

A previous study suggested equal treatment for equal need, regardless of socioeconomic status, for preterm children remaining within the control of the health service [9]. As such, socioeconomic status should not affect the care received in the neonatal period (lifesaving initial medical treatment, respiratory support, treatment of infections, adequate nutrition or length of stay) until first discharged from the hospital. This perspective was complemented by a study by Petrou and Kupek [13], who, using older data, found more hospital admissions, longer stays, and increased hospital costs for lower social classes, defined according to occupation in the general population. These authors highlighted that the effect of social class in the general population was most pronounced in the ages 3–10 years, compared to the first 2 years of life.

The aims of the current study were to describe the relationship between age and the use of hospital-based health care services in childhood for VPT children and to investigate the distribution of health care across five sets of characteristics, namely: child health, maternal health, social background, geography, and current age.

## Methods

### Study population and definitions

In this population-based registry study, we were interested in long-term follow-up on hospital use beyond the first year of life. Hence, children aged 1–9 years were included. We distinguished between outpatient visits and hospital admissions. Entry into the study was either January 1, 2008, or the date of the child’s first birthday, whichever came last. Follow-up extended to December 31, 2010, or the date of death, whichever came first. This allowed for follow-up from ages 1–9 years, with each child contributing a maximum of 3 years of follow-up, distributed across four age intervals. We included children born in Norway between January 1, 2001, and December 31, 2009, with a GA < 32 w or a BW < 1500 g, as registered in the Norwegian Medical Birth Registry (NMBR). A total of 9309 children met these inclusion criteria. Of these, we excluded 2881 stillbirths and 779 children who died before the date of entry into the study (*n* = 599), or after this date but before their first birthday (*n* = 180). A further 62 children, whose mothers had emigrated before January 1, 2008, were excluded, leaving 5587 children for inclusion into the study. Using a unique identification (ID) number, we linked information from the NMBR with information from the Norwegian Patient Register (NPR) and registries with information about sociodemographic characteristics of the mothers at Statistics Norway. Table 1 displays the follow-up by age in its most basic form. We accounted for different contacts at the same hospital and transfers between hospitals.

**Table 1:** Age-interval of children by birth year with follow-up in 2008-2010

Birth year	Number of unique children entering into study		Follow-up in 2008-2010			
2001	650					
		Age interval	6/7	7/8	8/9	9/10
		Person-years	318	648	648	327
2002	634					
		Age interval	5/6	6/7	7/8	8/9
		Person-years	313	630	629	318
2003	645					
		Age interval	4/5	5/6	6/7	7/8
		Person-years	323	643	641	317
2004	609					
		Age interval	3/4	4/5	5/6	6/7
		Person-years	302	607	606	302
2005	606					
		Age interval	2/3	3/4	4/5	5/6
		Person-years	292	606	604	312
2006	575					
		Age interval	1/2	2/3	3/4	4/5
		Person-years	285	571	568	284
2007	650					
		Age interval		1/2	2/3	3/4
		Person-years		651	643	314
2008	611					
		Age interval			1/2	2/3
		Person-years			612	303
2009	607					
		Age interval				1/2
		Person-years				297

The study builds upon the EuroHOPE comparison study of seven European countries for mortality and length of hospital stay for VPT children in their first year of life [23, 24] and utilizes Norwegian registry data on the complete population of VPT children. The Norwegian health care system is defined by low out-of-pocket payments and universal health care access that, in principle, does not depend on a household’s financial resources.

### Data

The NPR data contained information on the time of admission and discharge; emergency or planned admission; International Classification of Diseases, 10th revision (ICD-10 final diagnoses); and data on medical and surgical procedures. From the NMBR, we classified children according to their year of birth, GA in weeks (in categories: 22–24, 25–26, 27–28, 29–30 and 31+ w), sex, Apgar score at 5 min, multiple birth (yes/no), first in multiple birth, parity, and according to ‘the presence or absence of a congenital malformation (ICD-10 Q-chapter diagnosis). We defined small for gestational age status as birth weight below the first decile for (each week of) gestational age and sex, according to national tables [25]. We included measures of maternal health and complications during pregnancy, such as chronic hypertension, bleeding any time in pregnancy, HELLP syndrome, and any previous abortion prior to week 23.

We included a range of variables indicating the social and demographic background of the children. These included the civil status of parents at birth as recorded in the NMBR (married or cohabiting vs. alone [single, separated, or divorced]); and maternal characteristics: age at birth (NMBR if missing from Statistics Norway) and immigration status (first-generation immigrant or both parents immigrants) as defined by Statistics Norway. On social background we included maternal characteristics as level of education attained 2 years preceding the birth, income in the year preceding the birth and the education level of grandparents, using the highest level attained by either grandparent. Information on the father was limited and entered only through the civil status of the mother and paternal age.

We were interested in immigration status as it is a sociodemographic measure that is becoming increasingly prevalent among the population of VPT children. Over the period from 2001 to 2009, the percentage of immigrant mothers increased from 11% to 24%'among the mothers of very premature infants in Norway. We used the attained education level of maternal grandparents as a measure of underlying socioeconomic status, preferring it to maternal education level since the latter may well depend on the care outcomes of children (current or previous) in this population [26]. That is, giving birth or the health of a VPT child may interfere with attained education level. We argue that this is structurally not the case for the education of maternal grandparents, which we argue is exogenous to child health, but can be seen as an indicator of the socioeconomic support of the mother, and, by extension, the child.

We included the region of residence at birth, and the travel time from the home municipality to the nearest hospital to adjust for differences in outcomes across Norway’s hospital regions (Northern, Central, Western, and South-Eastern health authorities). These regions, responsible for specialized health care services within their catchment areas, have large differences in travel time to the nearest hospital. As living further away may result in parents substituting hospital care for care in other sectors, we included travel time by car to the nearest hospital from the home municipality in the year of birth (as calculated by Google Maps).

### Statistical analysis

To establish a comparison to the general population of Norwegian children, we used the definitions and grouping by ages 1-4 and 5-9 from Statistics Norway [27] to study the incidence and length of hospital admissions for VPT children that could be directly compared with official statistics. Due to the study design of follow-up in 2008-2010 (Table 1) and the study’s sample period of birth years 2001-2009 the comparison with the figures from Statistics Norway could only be produced for 2010. The figures from Statistics Norway were calculated with age defined crudely as calendar year minus birth year. However, since we for VPT children had access to information on both the date of birth and the date of the specific hospital contact we made use of the more detailed definition of current age, i.e. irrespective of calendar year, for subsequent calculations for this population. Embedded into this definition of current age is the concept of time at risk, calculated as the sum of time we observe each VPT child at a specific age.

To investigate trends by current age and GA as measures of child health we thereafter tabulated the sum of events over the time at risk to give the incidence rates of admissions and outpatient visits. For trends by current age and GA we also assessed the reason for the hospital contact by the proportion of care pertaining to each ICD-10 chapter.

Next, we assessed the relation of care to the characteristics described above separately for the age groups 1–4 and 5–9 years in multivariate regression models. For this purpose we used Poisson regression [28], summing the number of events as the outcome and the time at risk observed over each one-year of age as the offset. As the unit of observation was the child we adjusted the standard errors for multiple age-specific observations within each age interval, 1-4 and 5-9. The choice of the age-intervals 1-4 and 5-9 was based on the categorization of age in Statistics Norway [27], since this scheme would allow a simple investigation of the development over age by the various characteristics included on child health, maternal health, social background, and geography. We chose an inclusive variable selection strategy since the study is exploratory of these relationships. We judged that measures of social background established before or at a young age of the mother, such as immigration status and the educational level of grandparents, were of importance when considering confounding and should be included even if insignificant, and that this strategy should apply also to variables indicative of geography, i.e. belonging to a specific hospital region or travel time to hospital, as differences by these could be due to substitution of hospital care for care in other care providers. Other characteristics were subject to exclusion if insignificant by the Wald test at the 10% level in either model. We present the results as incidence rate ratios (IRRs) for a particular characteristic (i.e., boy) over the absolute incidence rate described by the intercept term of the reference category (i.e., girl), expressed per 1000 children per year.

All statistical analyses were performed using the *poisson* command in Stata (Release 13. College Station, TX: StataCorp LP.).

### Missing data

An overview of the study data is provided in Table 2. We had missing data in the resulting linked dataset on a small percentage of cases for a variety of variables (maximum 15% for a single variable). Birth weight and gestational age were partially missing, as was information pertaining to sociodemographic characteristics, such as educational attainment. To retain the full population in the regression analyses, we used multiple imputation of missing values. The multiple imputation procedure uses model-based estimates of uncertainty, allowing the resulting datasets to retain statistically valid variability [29]. Estimation in Stata proceeded with the prefix *mi estimate: poisson*. Further information on missing values and the imputation method used for each variable is shown in the Additional file 1: Table S1, S2 and S3].

**Table 2:** Descriptive statistics

Measure		Statistics	Person- years of observation
	Number of VPT Children	5587	13,915
	Mean (sd) Birth year	2005 (2.6)	13,915
Sex	# (%) Boy	2881 (51.6%)	7136
Gestational age	# (%) 22–24 weeks GA	198 (3.7%)	495
	# (%) 25–26 weeks GA	533 (9.9%)	1335
	# (%) 27–28 weeks GA	1029 (19.1%)	2565
	# (%) 29–30 weeks GA	1716 (31.8%)	4298
	# (%) 31+ weeks GA	1922 (35.6%)	4725
	# (%) Missing GA^a^	189 (3.4%)	498
Birth weight	Mean (sd) Birth weight	1275.6 (378.8)	13,592
	# (%) Missing birth weigh^a^	115 (2.1%)	323
	# (%) Small weight for GA	1575 (29.2%)	3875
	# (%) Missing weight for GA^a^	201 (3.6%)	534
Malformation at birth	# (%) Has Q-diagnosis	1183 (21.2%)	2903
Apgar score at 5 min	Mean (sd) Apgar score 5 min	8.3 (1.7)	13,592
	# (%) Missing Apgar score 5 min^a^	80 (1.4%)	323
Multiple birth	# (%) Multiple birth	1658 (29.7%)	4151
	# (%) First in multiple birth	4718 (84.4%)	11,726
Parity	# (%) No previous children	2972 (55.3%)	7268
	# (%) 1 previous child	2405 (44.7%)	6042
	# (%) Missing parity	210 (3.8%)	605
Age of mother	Mean (sd)	30.5 (5.5)	13,915
Health status of mother	# (%) Chronic hypertension	124 (2.2%)	302
	# (%) Bleeding any time in pregnancy	798 (14.3%)	1937
	# (%) Hellp syndrome	197 (3.5%)	500
	# (%) Any previous abortion	1323 (23.7%)	3296
Highest education	# (%) Primary school	851 (17.9%)	2199
among maternal grandparents	# (%) Secondary education	2765 (58.1%)	6937
	# (%) University education	1139 (24.0%)	2824
	# (%) Missing grandparents education level^a^	832 (14.9%)	1955
Immigration status	# (%) Non-Norwegian native	935 (16.7%)	2184
Civil status (at birth)	# (%) Single/separated/divorced	525 (9.5%)	1218
	# (%) Married/Cohabiting	4994 (90.5%)	12,521
	# (%) Missing civil status^a^	68 (1.2%)	175
Mothers’ education	# (%) Primary school	1265 (24.7%)	3159
(2 years before birth)	# (%) Secondary education	1949 (38.1%)	4982
	# (%) University education	1908 (37.3%)	4678
	# (%) Missing education^a^	465 (8.3%)	1096
Income (1 year before birth)	Mean (sd) in thousands	290.4 (205.7)	13,424
	# (%) Missing income^a^	206 (3.7%)	491
Age of father (at birth)	Mean (sd)	33.5 (6.7)	13,703
	# (%) Missing age of father^a^	92 (1.6%)	212
Geography (at birth)	Mean (sd) Travel time to hospital	24.9 (32.5)	13,915
Hospital region (at birth)	# (%) South-Eastern health authority	3059 (54.8%)	7581
	# (%) Western health authority	1254 (22.4%)	3098
	# (%) Central health authority	754 (13.5%)	1930
	# (%) Northern health authority	520 (9.3%)	1307

*VPT* very preterm, *sd* standard deviation, *GA* gestational age

Numbers in column ‘Statistics’ shows means and standard deviations (sd) for continuous variables, and the number of observations (#) and percentages (%) for categorical variables and non-missing observations

^a^For variables with missing values the number # and percentages (%) out of 5587 VPT children is shown. Person-years of observation is in the period 2008-2010

## Results

### Descri**p**tive statistics of study cohort

Over the period 2001–2009 in Norway, 1.22% of all live births were VPT (95% confidence interval (CI) 1.19–1.25%).^1^ However, 45% (CI 42-47%)^2^ of all deaths within the first year of life occurred in VPT infants. The average number of VPT children included in our database was 621 per year, and the 5587 children included for analysis in 2008–2010 were followed for an average of 2.5 years. Outpatient visits outnumbered hospital admissions by a ratio of 10:1.

### General population comparison

**Fig. 1:**
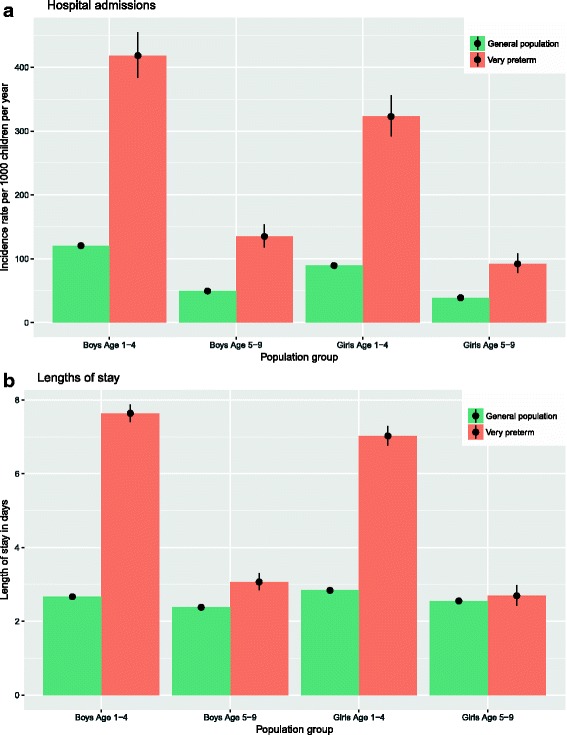
General population comparison of hospital admissions and lengths of stay (**a**, **b**)

In Fig. 1 [see also Additional file 1: Table S4], we compare the 2010 hospitalization rate and lengths of stay by VPT status in the general population [27]. VPT children had both admission rates and lengths of stay that exceeded those of children in the general population. Admissions differed by an IRR of 3.2 (CI 3.0–3.3, absolute rate comparison 227 vs. 72 hospitalizations per 1000 children) and the duration of admission among children born VPT was, on average, 2.3 times longer (CI 2.2–2.4, absolute length comparison 6.1 vs. 2.6 days per stay). The difference in admissions rates came closer in the older age group 5-9 years, however was still statistically significant by the 95% CIs. In the older age group, there was a significant increase in lengths of stay for boys, but not for girls.

### Age- and GA-specific rates

**Fig. 2:**
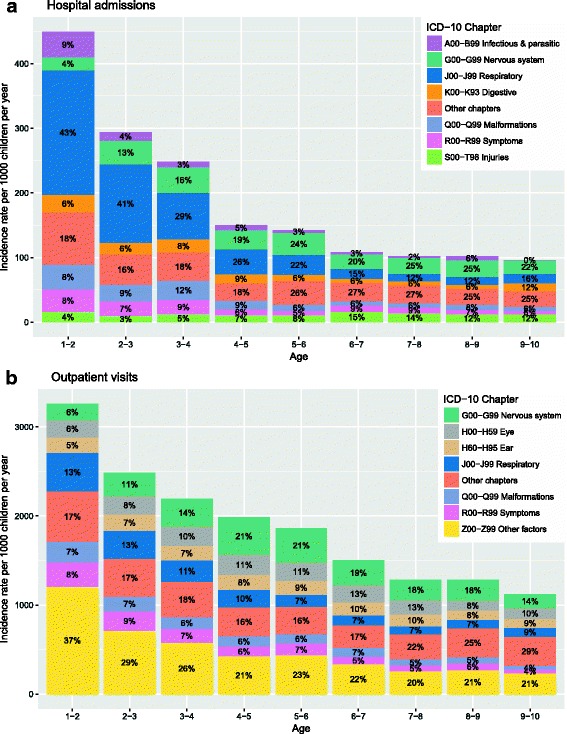
Age-specific rates per 1000 children per year for hospital admissions and outpatient visits (**a**, **b**)

The age-specific admission and outpatient visits rates for VPT children according to the reason for hospital contact (ICD-10 code) are given in Fig. 2. Tabulations according to ICD-10 chapter were restricted (due to data scarcity) to chapters accounting for more than 5% of the overall number of hospital contacts. Overall, the age-specific rates of hospital admissions and outpatient visits decreased steadily with age. Within each type of hospital care, there were exceptions to the overall pattern, as given by the percentages of the total age-specific rates. For example, the rates of hospital admissions vs. outpatient visits for disorders of the nervous system peaked in the 3- vs. 4-year-old age groups, respectively. Diseases of the respiratory system, nervous system, and malformations accounted for most of the 2831 hospital admissions. Diseases of the nervous system, respiratory system, and the eye accounted for the majority of the 28,036 outpatient visits. A large proportion (28%) of outpatient visits was related to general examinations.

**Fig. 3:**
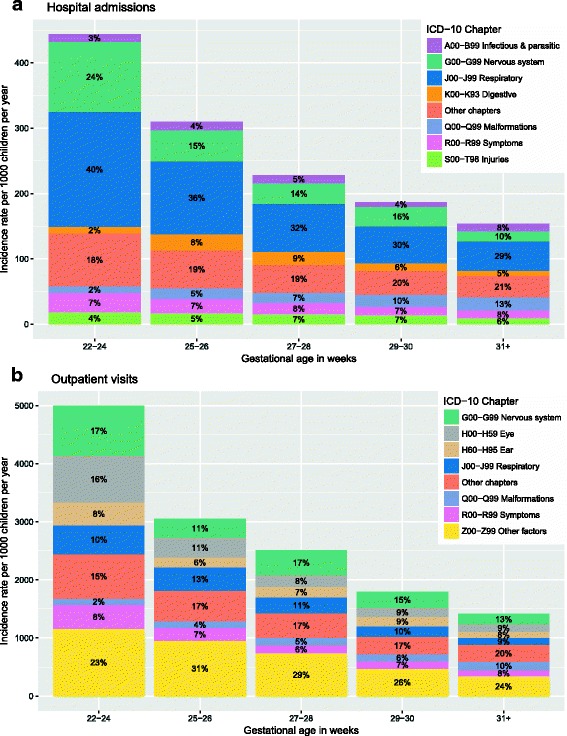
Gestational age-specific rates per 1000 children per year for hospital admissions and outpatient visits (**a**, **b**)

Figure 3 displays the GA-specific rates. For hospital admissions, a clear pattern was detected, with GA being associated with overall admissions, and particularly with admissions related to diseases of the nervous and respiratory systems. For outpatient visits, this pattern extended to diseases of the ears and eyes.

### Regression results

The regression results for the outcomes are presented in Table 3. Beyond travel time and immigration status, which we deemed adjustment factors, we excluded variables without any significant contribution in the final models. We excluded paternal age, maternal chronic hypertension or HELLP syndrome, variables on multiple birth and birth order, and maternal education and income. The highest education level among the maternal grandparents was usefully represented; we compared university education to secondary and primary school education combined. The results revealed that male sex, lower GA, presence of malformations at birth, and lower Apgar scores at 5 min, all increased the incidence of hospital admissions and outpatient visits across both age groups (1–4 and 5–9 years). As an example: for each 1-point increase in Apgar score at 5 min, the incidence of an admission between 1 and 4 years of age decreases by 7% (IRR 0.93, CI 0.89–0.97). Children small for their gestational age had excess hospital admissions between 1 and 4 years of age; 1.87 times higher (CI 1.56–2.24). This effect remained positive, but became insignificant for the ages 5–9 (IRR 1.15, p 0.38). We observed mixed results for maternal age, parity, bleeding at any time in pregnancy, and any previous abortion. The observed decrease in admissions associated with maternal age in children 1–4 years old (IRR 0.91 per 5-year increase, CI 0.85–0.98) should be weighed against the inclusion of parity, bleeding, and previous abortions, which are also included in the model. Upon removal of these, the effect of maternal age became insignificant and came closer to unity. We noted mixed results for geography and social background. VPT children in the older age group with grandparents whose highest education level was university had lower use of hospital admissions (IRR 0.72, CI 0.53–0.99) and outpatient visits (IRR 0.82, CI 0.69–0.97).

**Table 3:** Incidence rate ratios by ages 1–4 and 5–9 years

	Age 1–4					Age 5–9				
	(*n* = 4301)					(*n* = 3137)				
Hospital admissions	IRR	S.E.	P**	z	95%CI	IRR	S.E.	P**	z	95%CI
Boy (vs girl)	**1.22**	**0.10**	**0.014**	**2.47**	**1.04–1.44**	**1.38**	**0.19**	**0.017**	**2.38**	**1.06–1.80**
22–24 weeks GA (vs 31+)	**2.60**	**0.58**	**<0.001**	**4.28**	**1.68–4.03**	**2.34**	**0.75**	**0.008**	**2.67**	**1.25–4.37**
25–26 weeks GA (vs 31+)	**1.79**	**0.25**	**<0.001**	**4.22**	**1.37–2.35**	**1.52**	**0.35**	**0.070**	**1.81**	**0.97–2.39**
27–28 weeks GA (vs 31+)	**1.38**	**0.16**	**0.007**	**2.69**	**1.09–1.74**	**1.51**	**0.31**	**0.046**	**1.99**	**1.01–2.27**
29–30 weeks GA (vs 31+)	**1.41**	**0.15**	**0.002**	**3.15**	**1.14–1.74**	1.04	0.21	0.836	0.21	0.70–1.54
Small weight for GA (vs non-SWGA)	**1.87**	**0.17**	**<0.001**	**6.84**	**1.56–2.24**	1.15	0.18	0.384	0.87	0.84–1.57
Malformation at birth (vs absence)	**2.16**	**0.19**	**<0.001**	**8.52**	**1.81–2.57**	**1.55**	**0.22**	**0.002**	**3.04**	**1.17–2.05**
Apgar score at 5 min (difference from score 9)*	**0.93**	**0.02**	**0.001**	**−3.40**	**0.89–0.97**	0.94	0.05	0.187	−1.32	0.85–1.03
Parous (vs non-parous)	1.18	0.10	0.056	1.91	1.00–1.40	0.76	0.11	0.053	−1.93	0.58–1.00
Age of mother at birth (per 5 years from avg.)*	**0.91**	**0.03**	**0.010**	**−2.56**	**0.85–0.98**	1.03	0.06	0.579	0.55	0.92–1.16
Bleeding any time in pregnancy (vs none)	**1.31**	**0.14**	**0.012**	**2.51**	**1.06–1.63**	1.11	0.21	0.581	0.55	0.76–1.62
Any previous abortion up to 23rd week (vs none)	**1.27**	**0.12**	**0.010**	**2.58**	**1.06–1.52**	1.12	0.17	0.450	0.75	0.83–1.51
University education grandparents (vs < university)	1.12	0.11	0.259	1.13	0.92–1.36	**0.72**	**0.12**	**0.042**	**−2.03**	**0.53–0.99**
Mother is immigrant (vs native)	0.99	0.11	0.907	−0.12	0.79–1.23	0.97	0.18	0.882	−0.15	0.68–1.40
Single/separated/divorced (vs cohabiting/married)	0.84	0.11	0.176	−1.35	0.66–1.08	0.54	0.21	0.103	−1.63	0.25–1.13
Travel time hospital at birth (per 30 min from avg.)*	1.02	0.05	0.713	0.37	0.93–1.11	1.02	0.06	0.723	0.36	0.91–1.14
Western health authority (vs South-Eastern)	**0.73**	**0.08**	**0.002**	**−3.08**	**0.59–0.89**	0.95	0.19	0.807	−0.24	0.64–1.41
Central health authority (vs South-Eastern)	0.94	0.10	0.603	−0.52	0.76–1.17	1.10	0.22	0.642	0.46	0.74–1.62
Northern health authority (vs South-Eastern)	0.82	0.11	0.157	−1.42	0.62–1.08	**1.72**	**0.37**	**0.011**	**2.54**	**1.13–2.62**
Reference* rate per 1000 children per year	**181**	**44**	**<0.001**	**21**	**112–293**	**60**	**22**	**<0.001**	**11**	**29–123**
Outpatient visits	IRR	S.E.	P**	z	95%CI	IRR	S.E	P**	z	95%CI
Boy (vs girl)	**1.15**	**0.06**	**0.015**	**2.43**	**1.03–1.28**	**1.28**	**0.10**	**0.001**	**3.34**	**1.11–1.48**
22–24 weeks GA (vs 31+)	**3.12**	**0.44**	**<0.001**	**8.16**	**2.37–4.10**	**3.13**	**0.52**	**<0.001**	**6.82**	**2.26–4.35**
25–26 weeks GA (vs 31+)	**1.89**	**0.18**	**<0.001**	**6.72**	**1.57–2.27**	**2.05**	**0.27**	**<0.001**	**5.52**	**1.59–2.65**
27–28 weeks GA (vs 31+)	**1.68**	**0.14**	**<0.001**	**6.16**	**1.42–1.98**	**1.79**	**0.19**	**<0.001**	**5.43**	**1.45–2.20**
29–30 weeks GA (vs 31+)	**1.34**	**0.11**	**<0.001**	**3.65**	**1.15–1.57**	**1.31**	**0.14**	**0.011**	**2.54**	**1.06–1.62**
Small weight for GA (vs non-SWGA)	**1.50**	**0.09**	**<0.001**	**6.59**	**1.33–1.69**	**1.28**	**0.10**	**0.002**	**3.08**	**1.09–1.50**
Malformation at birth (vs absence)	**1.57**	**0.10**	**<0.001**	**7.18**	**1.39–1.77**	**1.53**	**0.13**	**<0.001**	**5.03**	**1.30–1.81**
Apgar score at 5 min (difference from score 9)*	**0.93**	**0.01**	**<0.001**	**−4.28**	**0.91–0.96**	**0.94**	**0.03**	**0.018**	**−2.36**	**0.89–0.99**
Parous (vs non-parous)	1.02	0.06	0.793	0.26	0.90–1.15	0.93	0.08	0.348	−0.94	0.79–1.09
Age of mother at birth (per 5 years from avg.)*	1.00	0.02	0.946	−0.07	0.95–1.05	1.05	0.04	0.131	1.51	0.98–1.13
Bleeding any time in pregnancy (vs none)	1.04	0.08	0.598	0.53	0.89–1.22	1.01	0.12	0.924	0.10	0.80–1.27
Any previous abortion up to 23rd week (vs none)	**1.17**	**0.08**	**0.025**	**2.25**	**1.02–1.34**	1.00	0.09	0.955	0.06	0.85–1.19
University education grandparents (vs < university)	1.09	0.08	0.211	1.25	0.95–1.25	**0.82**	**0.07**	**0.022**	**−2.28**	**0.69–0.97**
Mother is immigrant (vs native)	0.89	0.07	0.111	−1.59	0.76–1.03	0.94	0.11	0.587	−0.54	0.74–1.18
Single/separated/divorced (vs cohabiting/married)	0.98	0.10	0.873	−0.16	0.81–1.20	**0.76**	**0.10**	**0.042**	**−2.03**	**0.58–0.99**
Travel time hospital at birth (per 30 min from avg.)*	0.97	0.02	0.160	−1.40	0.92–1.01	0.95	0.03	0.079	−1.75	0.89–1.01
Western health authority (vs South-Eastern)	0.92	0.06	0.227	−1.21	0.80–1.05	1.15	0.11	0.153	1.43	0.95–1.38
Central health authority (vs South-Eastern)	1.04	0.08	0.624	0.49	0.89–1.21	**1.32**	**0.12**	**0.003**	**3.00**	**1.10–1.59**
Northern health authority (vs South-Eastern)	0.96	0.10	0.719	−0.36	0.79–1.18	**1.39**	**0.23**	**0.046**	**1.99**	**1.01–1.93**
Reference rate per 1000 children per year	**1133**	**192**	**<0.001**	**41**	**812–1579**	**502**	**115**	**<0.001**	**27**	**320–786**

*IRR* incidence rate ratio, *S.E.* standard error, *P p*-value, *z* z-value, *CI* confidence interval, *avg*. average, *GA* gestational age, *SWGA* small for gestational age

^*^Continuous variables Apgar score 5 min set to median score of 9, travel time and mothers age centered to overall mean

^**^Numbers in bold are significant at 5% level

Estimates are with multiple imputation of missing values

## Discussion

The results of this study add to the previous literature in several respects. Nordermoen and Bratlid [2] calculated the cost of treating premature children in Norway during the initial hospital admission. We added to their results by providing knowledge about hospitalizations beyond the first life year, and consistently demonstrated that this result is more pronounced for lower gestational ages. In particular, we found that children born in the gestational weeks 22-24 and 25-26, as well as children small for their gestational age and with a diagnosis of a congenital malformation (ICD-10 section Q); have excess hospital use. In contrast to the clinical characteristics of the child, characteristics indicative of maternal health, sociodemographics, and geographical proximity to hospital services had weaker and less consistent links with hospital admissions and outpatient visits. We similarly built upon an established methodology for comparing the care outcomes for VPT children in their first year of life across countries [23, 24].

We motivated the use of imputation as a method that allowed us to retain a perspective fully compatible with a population-based study. We chose imputation as our strategy to deal with missing data on the basis that, if the variables included are predictive of missing values, then the multiple imputation procedure should aid in correcting biases and preventing the loss of efficiency that would be encountered in a complete-case analysis [29].

Other previous studies have examined utilization of hospital services beyond the initial admission [10–20]. The present article contributes to the literature by a unique combination of register data and study design that allows us to perform a nationwide analysis of children aged 1–9 years born between January 1, 2001 and December 31, 2009. We adjusted our estimates of hospital care utilization for several measures, including maternal health, geographical access to hospital care, and socioeconomic status. Previous studies have analyzed data from specific districts, data from the 1970s and 1980s, or data covering fewer years beyond birth, or a combination of these characteristics. An extension to several previous studies is providing estimates for lower brackets of GA, and not limiting the results to a general finding for those delivered before 28 weeks. Our findings support the results of previous studies showing that clinical characteristics such as low GA and low BW contribute to hospitalizations beyond the initial admission, and that excess utilization of hospital resources on average declines with increasing age. In particular, prior to this study, hospital utilization during the period between 5 and 9 years old has been poorly documented. Similar to the study Petrou and Kupek [13], we found an increase in the effect of socioeconomic status on hospital utilization in later ages. As for immigration status, we did not uncover any effect on hospital use associated with non-native status. Disregarding the possibility of differences in cultural preferences for care at hospitals versus in primary care, this finding rejects a problem of access in case of under-utilization or problems of excessive comorbidity in the event of additional utilization compared with the native population. Acknowledging that immigration status covers heterogeneous groups we separated the growth of non-Norwegian status into that observed for mothers of European descent (increasing from 3% to 11%) and that observed for mothers of non-European descent (from 8% to 15%). This distinction emphasizes that compared to native Norwegians; both origins became more prevalent over the period considered, but that the increase was proportionally larger for mothers of European rather than for mothers of non-European descent. This situation accords with the overall development for Norway where the main increases in immigration in the past decade have come from countries in Eastern Europe.

We have documented variation in the utilization of health care resources according to place of living at birth for a nationwide population of VPT children. A recent report outlined substantial geographical variation in the use of hospital care for the general population of Norwegian children aged 0-16 years [30]. The authors of this report noted that the variation in hospital use could not be accounted for by differences in health status or by the characteristics of the health care providers. Similarly, a pattern by geographical proximity was not an apparent explanation for the observed differences. To the extent that we have adjusted the use of hospital care across hospital regions for potential systematic differences by health status; we note similar differences in the current study. Thus, the reasons for variation across regions remain unaccounted for. A question is whether this result is due to differences in local attitudes toward viability at birth. In particular, restricting care to babies born before 25 weeks GA may affect resource use later in childhood. An indication of such local attitudes may present itself by differences in the rates of live births. The Norwegian Research Council held a consensus conference in 1998 on the issue of gestational age limits for treatment of extremely premature infants [31]. This conference concluded that offering treatment to infants at 23 weeks’ gestational age was considered experimental medicine; in weeks 24 to 25 treatment could be offered if in agreement with the parent’s wishes and after week 25 treatment should be offered unless contraindicated for medical reasons. Hence, both the hospital regions as administrative entities and the therein residing parents are possible sources of local attitudes to viability. To see if this is reflected in the data we present a table on live and total births by the age of gestation and hospital region. In keeping with the categorization of gestational age in the current study, the table has been limited to infants below and above 25 weeks of gestation. Since there are four regions, we test for differences in expected versus observed live births across these using a chi-square test [Additional file 1: Table S5]. We find evidence of a difference for babies born <25 weeks gestation; in particular, for the Central health authority, whose observed live births are beyond those expected for the country as a whole. This result may indicate the presence of local attitudes to viability both from the side of parents and hospital regions, but from the size of the affected population we are unlikely to observe the effects of such sorting for hospital use of children aged 1-9. In all, we identify 530 live births of a total 1124 births with GA <25 weeks. However, only 198 are present in our analysis sample, mainly due to the exclusion criteria that limits the sample to children surviving to age one. For children age 1-9 the <25 GA group accounts only for 3.7% of our sample (cf. Table 2). Thus, the reasons for any difference between the hospital regions can be expected to lie elsewhere. It is a limitation that we are unable to ascertain why this population varies according to hospital regions in the use of hospital care. Further studies are needed to investigate the reasons for the geographical variation in use of hospital care for children born VPT and the general population of children and young adults alike.

The study had some limitations. First, owing to the study design we are unable to examine differences in hospital admissions and outpatient visits between those born early and late in the 2000’s as potential cohort effects. Generally, mortality by age one among the very preterm remained stable over the years considered as assessed by our data however other differences in the health of these children by birth year may not be captured satisfactorily by mortality as an indicator. Second, although it is generally a good practice to model continuous variables as such, we chose to model continuous age in two categories, 1-4 years and 5-9 years. Our choice was motivated by providing results that are easy to interpret and conforming to what we perceive as common categorizations in the literature. A more flexible modelling of continuous variables with for example splines might have been preferred methodologically, but would also complicate a presentation of results and discussion of possible developments with age by other characteristics. In future work, we aim at introducing a more flexible modelling of continuous variables to our work. Third, only admissions and consultations at public hospitals were included. For hospital admissions this is not a problem, since all admissions for this group of patients are at public hospitals. However, a considerable proportion of outpatient consultations are provided by privately practicing physician specialists who contract with a regional health authority. Iversen and Kopperud [32] showed, based on general population survey data, that socioeconomic status is positively correlated with utilization of private specialists once self-assessed health status has been adjusted for. If this result is generalizable and applicable to the population of VPT children, we may have underestimated the effect of socioeconomic status on health care utilization in this study. The effect of socioeconomic status on hospital care utilization is worth a discussion. As a measure thereof, we used maternal grandparents’ education in preference to the education level of the mother. In our data, the education of the grandparents is explanatory of the mothers’ attained education when adjusted for age and parity of the mother in a separate regression model. (The prevalence of mothers with higher education increases 33 percentage points if the maternal grandparents are also highly educated (CI 30-36).) We made two observations in models equal to those presented in Table 3 when we included the mother’s attained education in addition to grandparents’ education or mother’s education only. First, the coefficients of grandparents’ education were slightly stronger when included alongside maternal education, exhibiting the same pattern and statistical significance for the older age group. Second, the coefficients of maternal education were small and insignificant for either age group. Thus, while the two indicators of socioeconomic status are related, it is maternal grandparents’ education that contributes to explaining the use of hospital-based care. In general, an association between parents’ socioeconomic status and children’s hospital utilization can be established through at least two channels. First, parents with high socioeconomic status may communicate more easily with health professionals and, hence, be able to obtain better treatment for their children. This effect has previously been found by Finnvold [33]. Second, children of parents with high socioeconomic status may have better health status, as found by Petrou and Kupek [13]. This may not have been accounted for fully in the register data we used. Better health may imply less need for health care. Our empiric result of no association between parents’ socioeconomic status and children’s hospital utilization in the younger and needier age group shows the total effect of the two channels, and is a weak basis for making normative judgments.

## Conclusions

The relevance of this nationwide study is both related to exposing the variables that impact on hospital utilization of VPT children throughout ages 1–9 years and to predicting future hospital utilization for this patient group. We demonstrated substantial differences in hospital care utilization across age groups and clinical factors, adjusted for a host of factors pertaining to geographic and social background, and maternal health. The added information from these latter factors, although limited, was somewhat more evident within the 5–9 year age group (for social background), and mixed for geography and maternal health. The use of hospital care for the population of VPT children remains higher than their non-VPT peers in the general population throughout the ages 1–9 years.

**Fig. 4:**
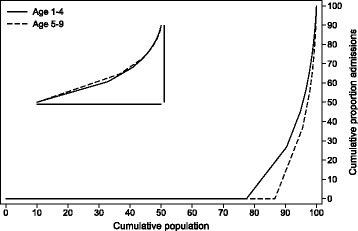
Lorenz curve of cumulative use of hospital admissions by proportion of population

**Fig. 5:**
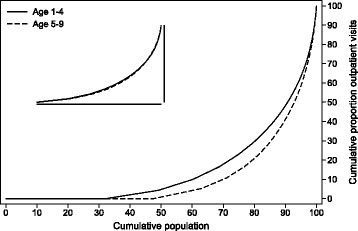
Lorenz curve of cumulative use of outpatient visits by proportion of population

Figures 4 and 5 show how care is distributed as a fraction of the population. The result was that the great majority of the children was healthy beyond the first year of life and did not require any particular follow-up from the hospital sector. Between 1 and 4 years of age, 22% of VPT children had at least one hospital admission. This reduced to 13% for 5–9 year-old children. As expected, inpatient admissions were more concentrated than outpatient consultations. This means that outpatient consultations were more evenly distributed among the children than inpatient admissions. Embedded into the graphs are figures of the distribution of use of the type of care for those with at least one hospital contact. These embedded figures demonstrate that, while fewer 5–9 year-old VPT children are in contact with the tertiary health care services, those who do remain have a similar distribution of care as in the 1–4 year age group.

## Additional file

Additional file 1: Use of hospital-based health care services among VPT children. (DOCX 35 kb)

## Abbreviations

BW: Birth weight
CI: Confidence interval
GA: Gestational age
NMBR: Norwegian Medical Birth Registry
NPR: Norwegian Patient Registry
SHR: Standardized hospitalization ratio
VPT: Very preterm

## Footnotes

1 The sum of live births from 2001 to 2010 in our database vs. total for Norway, 7102 vs. 583,045.

2 The sum of deaths by age 1 from 2001 to 2010 in our database vs. total for Norway, 828 vs. 1856.
