# Supplementary material for: Use of hospital-based health care services among children aged 1 through 9 years who were born very preterm - a population-based study
Source: BMC Health Serv Res. 2017 Aug 17;17:571. doi: 10.1186/s12913-017-2498-3 (PMC5561635; doi:10.1186/s12913-017-2498-3)
Supplement: Additional file 1: — Use of hospital-based health care services among VPT children. (DOCX 35 kb) [file 12913_2017_2498_MOESM1_ESM.docx]

# Appendix

**Multiple imputation methodology**

We included all covariates in all imputation models [1]. We used 30 imputations under a fully conditional specification for binary, categorical, and continuous covariates [2], and combined the results using Rubin’s rules [3]. Variables were imputed in wide format, separately for each age group. For binary and categorical variables we used logistic and multinomial logistic regression, whereas for continuous variables we used predictive mean matching. Predictive mean matching samples values from candidate donors (*n* = 10), so that the resulting values are realistic and lie within the range of observed values [4]. Beyond the general pattern of missing values noted in Table 2 of the main text, we were unable to trace a further 42 children and 43 mothers (1.5%) due to missing ID. While this prohibited linkage between registries, we still had information from the NMBR for this subset of children and mothers, from which we imputed missing information.

References

1. Sterne JA, White IR, Carlin JB, Spratt M, Royston P, Kenward MG, Wood AM, Carpenter JR. Multiple imputation for missing data in epidemiological and clinical research: potential and pitfalls. BMJ. 2009;338:b2393.
2. Van Buuren S. Multiple imputation of discrete and continuous data by fully conditional specification. Stat Methods Med Red. 2007;16:219–42.
3. Rubin DB. Multiple imputation for nonresponse in surveys. New York: Wiley; 1987.
4. Van Buuren S. Flexible imputation of missing data. Boca Raton: CRC press; 2012.

In Appendix Table S1 we note the imputation method for each variable with missing values.

#### Appendix Table S1. Imputation method

| **Measure** | **Method for imputation** |
| --- | --- |
| Gestational age | Predictive mean matching |
| Birth weight | Predictive mean matching |
| Apgar score 5 min | Predictive mean matching |
| Civil status (cohabitant or married vs. single, divorced or separated) | Logistic regression |
| Education level among maternal grandparents | Multinomial logistic regression |
| Education level of mother | Multinomial logistic regression |
| Previous number of children | Multinomial logistic regression |
| Age of father | Predictive mean matching |
| Income of mother | Predictive mean matching |
| Type of visit (admissions, outpatient)* | Predictive mean matching |
| Time in age interval* | Predictive mean matching |

*For children appearing in NMBR without a linkable ID-number.

In Appendix Table S2 we note the rates of admissions and outpatient visits for children who would have been in an complete-case analysis (never missing) and those excluded from such an analysis due to missing values on one or multiple characteristics (ever missing).

#### Appendix Table S2. Descriptive outcome statistics. Never missing. Rates per 1000 for children never missing

| Type of outcome | Age interval | 1–2 | 2–3 | 3–4 | 4–5 | 5–6 | 6–7 | 7–8 | 8–9 | 9–10 | # events |
| --- | --- | --- | --- | --- | --- | --- | --- | --- | --- | --- | --- |
| Admissions rate | | 462 | 301 | 242 | 143 | 139 | 108 | 90 | 108 | 93 | 2 104 |
| Outpatient visits rate | | 3 443 | 2 656 | 2 248 | 1 993 | 1 838 | 1 517 | 1 312 | 1 365 | 1 166 | 21 651 |
| Person-years | | 1 352 | 1 294 | 1 293 | 1 399 | 1 478 | 1 490 | 1 229 | 730 | 238 |  |

**Ever missing. Rates per 1000 for children ever missing**

| Type of outcome | Age interval | 1–2 | 2–3 | 3–4 | 4–5 | 5–6 | 6–7 | 7–8 | 8–9 | 9–10 | # events |
| --- | --- | --- | --- | --- | --- | --- | --- | --- | --- | --- | --- |
| Admissions rate | | 410 | 276 | 254 | 172 | 152 | 104 | 142 | 89 | 112 | 727 |
| Outpatient visits rate | | 2 756 | 2 060 | 2 044 | 1 957 | 1 951 | 1 457 | 1 201 | 1 030 | 1 008 | 6 385 |
| Person-years | | 493 | 515 | 496 | 420 | 396 | 402 | 366 | 236 | 89 |  |

#### In Appendix Table S3 we present statistics on missing values for each variable, differentiating the characteristics of the children who would have been included in an complete-case analysis (never missing) and those excluded from such an analysis due to missing values on one or multiple characteristics (ever missing). Appendix Table S3. Descriptive statistics by never missing and ever missing status

| **Measure** | **Never Missing** | **Ever missing** | **Difference** | p<\|z\| |
| --- | --- | --- | --- | --- |
|  | (R0) | (R1) | (R1-R0)* |  |
| Number of VPT Children | 4 222 | 1 365 |  |  |
| Mean (sd) birth year | 2 004.9 (2.6) | 2 005 (2.6) | 0.11 | 0.184 |
| # (%) Boy | 2 191 (51.9%) | 690 (50.5%) | -1.4% | 0.387 |
| # (%) 22–24 weeks GA | 136 (3.2%) | 62 (4.5%) | 1.3% | 0.035 |
| # (%) 25–26 weeks GA | 408 (9.7%) | 125 (9.2%) | -0.5% | 0.575 |
| # (%) 27–28 weeks GA | 812 (19.2%) | 217 (15.9%) | -3.3% | 0.004 |
| # (%) 29–30 weeks GA | 1 352 (32.0%) | 364 (26.7%) | -5.4% | <0.001 |
| # (%)31+ weeks GA | 1 514 (35.9%) | 597 (43.7%) | 7.9% | <0.001 |
| # Missing GA | 0 (0.0%) | 189 (13.8%) |  |  |
| Mean (sd) Birth weight | 1 283.6 (378.7) | 1 248.3 (378.2) | -35.3 | 0.004 |
| # Missing birth weight | 0 (0.0%) | 115 (8.4%) |  |  |
| # (%) Small weight for GA | 1 238 (29.3%) | 337 (29.0%) | -0.4% | 0.805 |
| # Missing weight for GA | 0 (0.0%) | 201 (14.7%) |  |  |
| # (%) Malformation at birth | 913 (21.6%) | 270 (19.8%) | -1.8% | 0.14 |
| Mean (sd) Apgar score 5 min | 8.3 (1.6) | 8.2 (1.8) | 0.0 | 0.425 |
| # Missing Apgar score 5 min | 0 (0.0%) | 80 (5.9%) |  |  |
| # (%) Multiple birth | 1 315 (31.1%) | 343 (25.1%) | -6.0% | <0.001 |
| # (%) First in multiple birth | 3 531 (83.6%) | 1 187 (87.0%) | 3.3% | 0.002 |
| # (%) No previous children | 2 391 (56.6%) | 581 (50.3%) | -6.3% | <0.001 |
| # (%) 1 child from before | 1 831 (43.4%) | 574 (49.7%) | 6.3% | <0.001 |
| # Missing parity | 0 (0.0%) | 210 (15.4%) |  |  |
| Mean (sd) Age of mother | 30.5 (5.3) | 30.5 (6) | 0.0 | 0.886 |
| # (%) Chronic hypertension | 94 (2.2%) | 30 (2.2%) | 0.0% | 0.95 |
| # (%) Bleeding any time in pregnancy | 628 (14.9%) | 170 (12.5%) | -2.4% | 0.021 |
| # (%) Hellp syndrome | 159 (3.8%) | 38 (2.8%) | -1.0% | 0.065 |
| # (%) Any previous abortion up to 23rd week | 1 008 (23.9%) | 315 (23.1%) | -0.8% | 0.544 |
| # (%) Grandparents Primary school | 744 (17.6%) | 107 (20.1%) | 2.5% | 0.18 |
| # (%) Grandparents Secondary education | 2 477 (58.7%) | 288 (54.0%) | -4.6% | 0.043 |
| # (%) Grandparents University education | 1 001 (23.7%) | 138 (25.9%) | 2.2% | 0.277 |
| # Missing grandparents educ. | 0 (0.0%) | 832 (61.0%) |  |  |
| # (%) Mother is immigrant | 141 (3.3%) | 794 (58.2%) | 54.8% | <0.001 |
| # (%) Single/separated/divorced | 336 (8.0%) | 189 (14.6%) | 6.6% | <0.001 |
| # (%) Married/Cohabiting | 3 886 (92.0%) | 1 108 (85.4%) | -6.6% | <0.001 |
| # Missing civil status | 0 (0.0%) | 68 (5.0%) |  |  |
| # (%) Mother Primary school | 987 (23.4%) | 278 (30.9%) | 7.5% | <0.001 |
| # (%) Mother Secondary education | 1 653 (39.2%) | 296 (32.9%) | -6.3% | <0.001 |
| # (%) Mother University education | 1 582 (37.5%) | 326 (36.2%) | -1.3% | 0.48 |
| # Missing education | 0 (0.0%) | 465 (34.1%) |  |  |
| Mean (sd) Income in thousands | 305.5 (205.1) | 235.4 (198.5) | -70.1 | <0.001 |
| # Missing income | 0 (0.0%) | 206 (15.1%) |  |  |
| Mean (sd) Age of father | 33.1 (6.3) | 34.8 (7.7) | 1.7 | <0.001 |
| # Missing age of father | 0 (0.0%) | 92 (6.7%) |  |  |
| Mean (sd) Travel time to hospital | 24.9 (31.1) | 24.7 (36.3) | -0.2 | 0.837 |
| # (%) South-Eastern health authority | 2 240 (53.1%) | 819 (60.0%) | 6.9% | <0.001 |
| # (%) Western health authority | 984 (23.3%) | 270 (19.8%) | -3.5% | 0.005 |
| # (%) Central health authority | 620 (14.7%) | 134 (9.8%) | -4.9% | <0.001 |
| # (%) Northern health authority | 378 (9.0%) | 142 (10.4%) | 1.5% | 0.121 |

VPT, very preterm; sd, standard deviation; GA, gestational age

*Binomial regression model for variables listed by # (%), linear regression for variables listed by Mean (sd).

In Appendix Table S4 we compare the rate of hospital admissions and lengths of stay with the general population of children.

***Appendix Table S4****. General population comparison of hospital admissions*

|  |  | Number of hospital admissions | Number of hospital days | Number of persons | Admissions per  1000 persons | Hospital days per admission |
| --- | --- | --- | --- | --- | --- | --- |
| **Girls** |  |  |  |  | Rate (95% CI) | Days (95% CI) |
| Ages 1–4 | Population | 10 435 | 29 599 | 116 733 | 89 (88–91) | 2.8 (2.8–2.9) |
|  | VPT | 380 | 2 669 | 1 177 | 323 (290–355) | 7.0 (6.8–7.3) |
|  |  |  |  |  |  |  |
| Ages 5–9 | Population | 5 628 | 14 350 | 144 545 | 39 (38–40) | 2.5 (2.5–2.6) |
|  | VPT | 139 | 374 | 1 512 | 92 (77–107) | 2.7 (2.4–3.0) |
| **Boys** |  |  |  |  |  |  |
| Ages 1–4 | Population | 14 782 | 39 404 | 122 706 | 120 (11–9 122) | 2.7 (2.6–2.7) |
|  | VPT | 522 | 3 987 | 1 248 | 418 (382–454) | 7.6 (7.4–7.9) |
|  |  |  |  |  |  |  |
| Ages 5–9 | Population | 7 454 | 17 723 | 150 800 | 49 (48–51) | 2.4 (2.3–2.4) |
|  | VPT | 218 | 668 | 1 616 | 135 (117–153) | 3.1 (2.8–3.3) |

VPT, very preterm; CI, confidence interval

Age defined as year of stay minus year of birth.

Source Population numbers from Statistics Norway adjusted for presence of VPT children. VPT numbers from Medical Birth Registry* minus emigres.

Hospital admissions, number of hospital days and number of persons, by gender, ages 1–4 or 5–9, and VPT status for the year 2010, not including admissions at psychiatric wards or admissions for patients without a valid municipality number,

In Appendix Table S5 we present statistics on live births and total births across Norway’s hospital regions.

#### Appendix Table S5. Live births vs. total births by gestational age (< 25, ≥ 25) and hospital region

| **Gestational age 22-24 weeks** | | | | | |
| --- | --- | --- | --- | --- | --- |
|  | Hospital region (health authority) | | | |  |
|  | South-Eastern | Western | Central | Northern | Total |
| Live births | 267 (44%) | 123 (48%) | 92 (56%) | 48 (49%) | 530 (47%) |
| Total births | 607 (100%) | 256 (100%) | 163 (100%) | 98 (100%) | 1 124 (100%) |
| P-value Gestational age 22-24 weeks= 0.04* | | | | | |
|  |  |  |  |  |  |
| **Gestational age ≥25 weeks** | | | | | |
|  | Hospital region (health authority) | | | |  |
|  | South-Eastern | Western | Central | Northern | Total |
| Live births | 3 157 (90%) | 1 272 (89%) | 737 (91%) | 527 (89%) | 5 693 (90%) |
| Total births | 3 520 (100%) | 1 427 (100%) | 810 (100%) | 589 (100%) | 6 346 (100%) |
| P-value Gestational age ≥25 weeks= 0.58* | | | | | |

Note: Stillbirths can be calculated by subtracting live births from total births. The table only includes children with information on gestational age and gestational age in the interval 22 – 39 weeks or birth weight <1500g.

*The p-values are from two separate chi-square tests for a difference in observed versus expected frequencies of live births versus stillbirths across the hospital regions.
